# Dietary Behaviors and Biochemical Serum Indicators in Adults with Type 2 Diabetes Mellitus from a Rural Region of Jalisco, Mexico

**Published:** 2018-04

**Authors:** Monica NAVARRO-MEZA, Karina Anai GARCIA-CAMACHO, Felipe SANTOYOTELLES, Antonio LÓPEZ-ESPINOZA, Omar ARROYO-HELGUERA

**Affiliations:** 1. Molecular Biology Chronic Illness Research Center, South University Center, University of Guadalajara, Guadalajara, Jalisco, Mexico; 2. Nutrition and Nutritional Behavior Research Center, South University Center, University of Guadalajara, Guadalajara, Jalisco, Mexico; 3. South University Center, University of Guadalajara, Guadalajara, Jalisco, Mexico; 4. Ecology and Health Laboratory, Institute of Public Health, Universidad Veracruzana, Veracruz, México

## Dear Editor-in-Chief

This cross sectional study was about the dietary behaviors and biochemical serum indicators in patients with type 2 diabetes mellitus (T2DM) living in a rural area of Jalisco, México, in 2015–2016.

The methodological procedure was approved and accepted by the Ethics and Research Committee of the Sanitary Region VI of the Health Department State of Jalisco, Mexico.

T2DM is ranked as fourth leading cause of death in Mexico and is considered one of most important health public problems (1, 2). The increase of T2DM incidence rates is associated with eating habits and lifestyle (3).

Thirty-four patients’ adults with early T2DM were enrolled. Data were gathered from an eating behavior survey applied (4). We determined serum levels of glucose, triglyceride, total cholesterol, high-density (HDL), low-density lipoprotein cholesterol (LDL) insulin, interleukin-6 (IL-6) and total antioxidant capacity (TAC). The demographic and clinical characteristics are in Table 1; the glucose values were related to avoiding certain foods due to their dislike (*P*=0.037); cholesterol values were related to a dislike of sea food and fish (*P*=0.04); as well as with habit of not usually including dessert i n main meal (*P*=0.04) and having sweetened fruit (*P*=0.028) and non-sweetened fruit (*P*=0.002); LDL was related to behavior at moment of selecting food based on the analysis of the nutritional content by the patient (*P*=0.025) and liking nuts, almonds, pistachios was associated with HDL; triglyceride levels were associated with behaviors such as choosing food by its appearance (*P*=0.005); drinking fresh water during day (*P*=0.014): including soup or any other entry usually (*P*=0.026) tortilla, bolillo roll or tostadas (*P*=0.013)*.* Insulin levels were associated with following eating behaviors: also in those cases were another person was in charge of preparing the meals frequently during week (*P*=0.01), chewing each bite for more than 25 times (*P*=0.006), liking a great deal fruits (*P*=0.002); vegetables (*P*=0.009); beans, chickpeas and lentils (*P*=0.039) eggs (*P*=0.045), having usually fruits or vegetables (*P*=0.001) and with including food during the main meal (*P*=0.031); IL-6 levels were also associated with having another person prepare their meals with a major frequency during week (*P*=0.01); chewing more than 25 times (*P*=0.006), showing a n indifference towards fruits (*P*=0.012) and not including fruit in their main meal (*P*=0.006); TAC was associated with liking a great deal, almonds, nuts (*P*=0.001).

**Table 1:** Demographic and clinical characteristics in adults with Type 2 Diabetes Mellitus

***Characteristics***	***n= (%)***	***Biochemical serum indicators***	***n= (%)***
Age(yr), Mean± SD	49.94 ± 10.64	Glucose, mg/dl, mean±SD	173.09 ± 74.69
Women	25 (74)	Glucose, mg/dl, <60	0
Men	9 (26)	Glucose, mg/dl, 60–110	3 (10)
BMI, (kg/m^2^) Mean± SD	29.63 ± 4.40	Glucose, mg/dl, >110	28 (90)
Low weight	0	Total cholesterol, mg/dl, mean±SD	174.04 ± 41.63
Normal weight	6 (18)	Total cholesterol, mg/dl, <200	21 (67.7)
Overweight	15 (44)	Total cholesterol, mg/dl, 200–239	8 (25.8)
Obesity type I	9 (26)	Total cholesterol, mg/dl, >249	2 (6.5)
Obesity type II	4 (12)	Cholesterol LDL, mg/dl, mean ± SD	106.50 ± 27.87
Civil status (Single)	5 (15)	Cholesterol LDL, mg/dl, <100	11 (32)
Civil status (Marriage)	22(64)	Cholesterol LDL, mg/dl, 100–129	14 (41)
Civil status (Widower)	3(9)	Cholesterol LDL, mg/dl, 130–160	6 (18)
Civil status (Divorced)	2(6)	Cholesterol HDL, mg/dl, mean ± SD	45.14 ± 11.23
Civil status (Free Union)	2(9)	Cholesterol HDL, mg/dl, Men>50	0
Education Primary	10 (29)	Cholesterol HDL, mg/dl, Men 35–50	1 (3)
Education Middle School	10 (29)	Cholesterol HDL, mg/dl, Men<35	8 (26)
Education High School	5 (15)	Cholesterol HDL, mg/dl, Women>60	0
Education Other	8 (24)	Cholesterol HDL, mg/dl, Women 45–60	15 (16)
Education No studies	1 (3)	Cholesterol HDL, mg/dl, Women<45	17 (55)
Physical activity (Yes)	13 (38)	Insulin, μU/ml, mean ± SD	54.75 ± 21.47
Physical activity (No)	21(62)	Insulin, μU/ml, 0.7–25	1 (3)
Comorbidities (Arterial hypertension)	13 (38)	Insulin, μU/ml, >25	29 (97)
Comorbidities (None)	21 (62)	Interleucin-6 (pg/ml)	499 ±116
Drug consumption <3	25 (74)	Antioxidant capacity (mM uric acid equivalent)	0.061±0.09
Drug consumption >3	1 (3)		
No Drug consumption	8 (23)		

Note: Drugs consumed are: metformin and glibenclamide, insulin, antihypertensives, acetylsalicylic acid, statins: atorvastatin, cinnarizine, anticonvulsant/antineuralgic: pregabalin, and non-steroidal anti-inflammatory

This study showed that level of income was a factor to help them improve eating behaviors. Nevertheless, lacks information, compromise or personal motivation were also considered as factors to improve their eating habits. The risk to lose the acquisition of a new habit can occur due to preferences and rejection to certain types of food (5) where the health status or illnesses of each individual can interfere.

The election and consumption of foods not prepared at home can be related to the habit of eating sugar-sweetened and processed foods and as a result, developments of metabolic alterations like obesity. In Mexico, people of lower socioeconomic status consume more sweetened beverages such as soft drinks. During the transition of eating habits, certain behaviors develop such as eating fast foods and processed foods which have a high content of refined sugars, saturated fats and salt, and evidence has shown that these behaviors occur more often in occidental countries in patients who are likely to suffer T2DM.

The importance of assessing eating behaviors in patients with T2DM and impact of food on progression of disease highlights the need for more research, especially to clarify and understand eating patterns in T2DM in rural regions. We suggest patients may improve their quality of life through an early intervention aimed at preventing comorbidities associated with T2DM.
